# 2D-Graph of intermolecular interactions predicts radical character of anion–π* type charge-transfer complexes †

**DOI:** 10.1039/d3ra07729b

**Published:** 2024-01-25

**Authors:** Zhenda Lin, Hao Su, Wenhuan Huang, Xuepeng Zhang, Guoqing Zhang

**Affiliations:** a Hefei National Research Center for Physical Sciences at the Microscale, University of Science and Technology of China Hefei 230026 China zhangxp@ustc.edu.cn gzhang@ustc.edu.cn

## Abstract

The molecular orbital (MO) theory is one of the most useful methods to describe the formation of a new chemical bond between two molecules. However, it is less often employed for modelling non-bonded intermolecular interactions because of the small charge-transfer contribution. Here we introduce two simple descriptors, the energy difference (*E*_DA_) of the HOMO of an electron donor and the LUMO of an acceptor against such HOMO–LUMO overlap integral (*S*_DA_), to show that the MO theory could give a unified charge-transfer picture of both bonding and non-bonding interactions for two molecules. It is found that similar types of interactions tend to be closer to each other in this 2D graph. Notably, in a transition region from strong bonding to single-electron transfer, the interacting molecular pairs appear to present a “hybrid” between chemical bonding and a radical pair, such as anion–π* interactions. It is concluded that the number of nodes in the HOMO and LUMO play a crucial role in determining the bonding character of the molecular pair.

## Abstract 2

An alternative description of bimolecular interactions in organic chemistry is proposed, where the overlap integral (*S*_DA_) and energy gap (*E*_DA_) between donor HOMO and acceptor LUMO are used as two axes in the 2D map.

## Graphical abstract

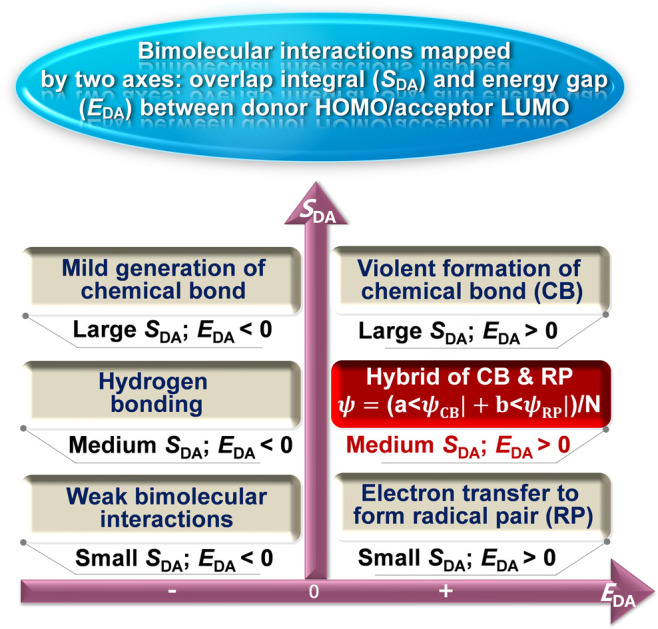

When two organic molecules are in close proximity to one another, many types of bimolecular interactions (BMIs) could happen, including (1) chemical reactions with new bond formation and old bond cleavage, (2) spontaneous single-electron transfer resulting in a pair of radicals,^1,2^ and (3) non-bonding interactions such as hydrogen and halogen bonds,^3,4^ π–π stacking^5,6^ or van der Waals forces. Despite all BMIs being coulombic in origin, definitions for such interactions are largely loose or intuitive with many overlapping characteristics that are difficult to separate. For example, there has been much debate on the force nature of the seemingly simplistic hydrogen bonding over the past century, with a wide range of dominating contributions such as electrostaticity, covalency (charge-transfer), polarization, and secondary electrostatic interactions.^7^ The difference in opinion is largely caused by the theoretical method used, various atomistic understandings are revealed by quantum or molecular mechanics models and calculations.^8–10^ In the discussion of BMIs, each method emphasizes differently on the contribution of forces and, therefore, non-arbitrary definitions of how to deconvolute contributions do not exist. Regardless of the contradictory conclusions, the theoretical results are still invaluable in informing or predicting many relevant areas of experimental chemistry.^9,11–13^

Molecular orbital (MO) theory is a useful and versatile tool for solving problems in organic chemistry, particularly in predicting the reactivity and site of chemical bond formation between two reactants.^14,15^ Compared with valance-bond theory, MO theory is more powerful since the geometries of the reactants can be inherently manifested and can thus be used to predict the extent of orbital overlap and thus the strength of interactions. In the MO theory, a reactant molecule is holistically viewed as one single united and distorted atom, in which a pair of electrons from an occupied molecular orbital (usually the highest, or HOMO) moves toward an unoccupied molecular orbital (usually the lowest, or LUMO) of another reactant to form (and sometimes break) a chemical bond and yield the product following the relocation of electrons. Empirically, whether or not a chemical bond can eventually form depends on both the spatial overlap and symmetry, and the energy difference, between the two interacting MOs. Referring to the idea in the screening of electro-fluorescent materials,^16^ the two descriptors can be defined as the following: the overlap integral *S*_DA_ = 〈*Φ*_D_|*Φ*_A_〉 and the calculated HOMO–LUMO energy difference *E*_DA_ = *E*_D_ − *E*_A_. For most molecular pairs, we assume that these two orbitals are predominately involved in the bonding process. For calculation purposes, it has to be noted that *S*_DA_ and *E*_DA_ can vary substantially based on the orbital (*e.g.*, HOMO *vs.* HOMO−1), reacting configuration and functional selection.^17^

Despite its productive applications, the MO method is not commonly used in describing non-bonding BMIs because of the small degree of covalency or charge transfer present in the systems. Instead, many excellent theories (*e.g.*, electrostatic, dipole, Marcus model,^18,19^*etc.*) have been developed to account for the observed phenomena such as cation–π, hydrogen bonding, and charge-transfer complexes. Here in this study, we explore the possibility of using the two MO descriptors, *S*_DA_ and *E*_DA_, to give a coherent description of the CT component for all types of BMIs. For stronger non-bonding interactions such as anion–π* interactions, the CT character could be significant enough to allow for reasonable prediction of the properties of the molecular pair, which leads to the motivation for interests in modelling this particular category: the structures of CT complexes are not directly observable from experiments and can only be deduced from rotational constants. An added layer of complexity is that CT complexes in solution are usually ephemeral, which suggests that experimental results could be highly finnicky. Finally, since the use of Hartree–Fock method is expected to miss the correlation energy, the optimized geometry is obtained with the density-functional theory (DFT) using Gaussian 16 software pack^20^ at B3LYP with empirical dispersion and 6-311+G(d, p) level; for consistency, the energy levels of HOMO and LUMO are calculated at the same level, where the overlap integral between the two MOs is calculated using the Multiwfn software.^21^ In our method, *S*_DA_ is specifically referred to as the overlap integral for the transition state for chemical reactions (since there exists multiple possible local minima for pre-reaction complexes) and electron transfer processes (for reactions without a transition state in cation–anion reactions, we simply tear the product molecule into two separate components); for non-bonding interactions, *S*_DA_ is calculated from energy-optimized (lowest energy) molecular configurations for the pair of molecules. Again, the study is only an attempt to provide additional theoretical insights into the CT character for weakly interacting molecules which are difficult to measure experimentally and is not meant to give accurate results energetically.

As can be seen from Fig. 1a, the majority of *S*_DA_ values for the transition state of a chemical reaction fall within the range of 0.65–0.20, which is not surprising since a chemical bond does require substantial initial energy gain from in-phase overlap to offset the energy cost on nuclear rearrangements. The energy difference *E*_DA_ in these limited examples of chemical reactions in vacuum falls between −5.26 eV (for slow reactions such as non-substituted Diels–Alder reactions) and +15.26 eV for reactions that are violent or essentially explosive (*e.g.*, *t*Bu^−^ and H^+^). In comparison, Fig. 1b shows examples of typically very weak non-bonding interactions such as CH_4_⋯CH_4_ (−11.02 eV), and strong ones like CH_3_NH_3_^+^…C_6_H_6_ (−1.14 eV). Not surprisingly, non-bonding pairs exhibit *S*_DA_ values are very small (*e.g.*, 0.065 for C_6_H_6_⋯C_6_H_6_). However, the non-zero values suggest that CT or covalency exists for all types of BMIs according to this method. The reason for a small *S*_DA_ is most likely a combination of longer average equilibrium distance (due to stronger Pauli repulsion) and/or bad symmetry match between frontier orbitals of the pair. It is interesting to notice that the *S*_DA_ is larger for two benzene molecules *vs.* two methane ones due to stronger C_6_H_6_⋯C_6_H_6_ attraction, which is quite consistent with the reality that benzene is more easily to form a condensed phase while methane is not, excluding considerations from other components such as electrostatic and multipole interactions. However, the *S*_DA_ value for the two benzene molecules is still significantly smaller than that of the chemical reaction because of out-of-phase cancellation of the overlapping orbitals despite a relatively close distance, which indicates that increased numbers of nodes in HOMO and LUMO reduce covalency. The “in-between cases” shown in Fig. 1a and b (*E*_DA_ < 0 and *S*_DA_ = 0.10–0.20) are presented in Fig. 1c, where *S*_DA_ is smaller compared to that of the bimolecular chemical reactions from Fig. 1a but larger than that of non-bonding pairs in Fig. 1b. Interestingly, we found that such interactions are exclusively hydrogen bonding in category. The results are consistent with some of the previous findings that covalency or CT plays a dominant role in hydrogen bonds.^22^ In this case, one orbital has a very small node number while the other is significantly larger, which allows for increased overlap integral and thus covalency. Fig. 1d shows an interesting class of interactions with positive *E*_DA_ and near zero *S*_DA_ values. These pairs appear to form what are known as ground-state “charge transfer complexes (CTCs)”, with a single electron spontaneously transferred from a donor to an acceptor and a pair of radicals generated.^23^ A positive *E*_DA_ suggests that thermal electron transfer is favorable with a low kinetic barrier from the Marcus model; again, since there tends to be more nodes in the frontier MOs in large π-conjugated molecules, it is unfavorable to form chemical bonds because the overall overlap is unlikely to due to many out-of-phase overlaps.

**Fig. 1:**
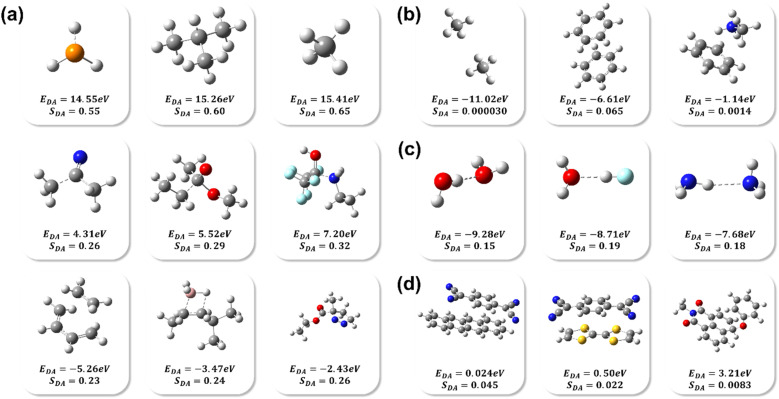
Optimized molecular configurations in the following bimolecular interactions: (a) PH_2_^−^ anion and proton, *tert*-butyl anion and proton, methyl anion and proton, acetyl cation and methyl anion, ethyl acetate and ethyl anion, ethylamine and acetone hexafluoride, ethylene and *cis*-butadiene, (*Z*)-4-methylpent-2-ene and borane, ethyl methacrylate and diazomethane; (b) methane and methane, benzene and benzene, benzene and methylammonium cation; (c) water and water, water and hydrogen fluoride, ammonia and ammonia; (d) tetracene and *p*-benzenedimalononitrile, tetrathiafulvalene and *p*-benzenedimalononitrile, *N*-methyl-1,8-naphthalimide and phenol anion.

As the trend becomes clear with more calculated pairs, we can plot a two-dimensional graph, shown in Fig. 2, using *E*_DA_ as the *X* axis and *S*_DA_ as the *Y* axis. If we arbitrarily divide the graph into six sections (which are labelled A1, A2, B1, B2, C1 and C2, respectively), they could then represent six different types of BMIs based on the contribution of the CT component and energy difference. Logically, A1 and A2 are hard and easy chemical reactions, respectively; interactions in C1 and C2 should correspond to non-bonding interactions and single-electron transfer, respectively. As a transition region from A1 to C1, B1 should in principle be filled with cases of “special bonding”, primarily hydrogen bonds. A borderline situation between a piperidine donor and a C_60_ acceptor (*E*_DA_ = −2.029 eV; *S*_DA_ = 0.104, Fig. S1†) indicates that such an interaction might be much stronger than van der Waals force but weaker than a conventional chemical bond. Indeed, the pair is recently examined by Hobza *et al.*^24^ and is concluded to be a dative bond. At this point, it is not difficult to notice that B2 is not populated with any commonly seen interactions. However, as a transition region from A2 to C2, B2 should mainly contain interactions exhibiting characters with both chemical bonding and electron transfer. Again, the number of nodes in the two orbitals exhibit a mismatch, which indicates a moderate degree of covalency. It is well-known that a radical pair (RP) is created post single-electron transfer.^1,2,25^ However, it must also be considered that cases in B2 should also exhibit covalency, *i.e.*, much higher stability than CT interactions. If we designate the RP state as *ψ*_RP_ and chemical bonding state as *ψ*_CB_, the hybrid B2 state should be described as *ψ*_Hybrid_ = *a* <*ψ*_RP_|+ *b* <*ψ*_CB_, where *a* and *b* are complex coefficients. Therefore, B2 depicts a peculiar state that is more localized than conventional RPs but exhibits substantial CT or diradical character than conventional chemical bonding; the superimposed state can yield either outcome during measurement (*e.g.*, with only a percentage of population exhibiting detectable unpair electron signals while the rest of the pair stays spectroscopically dormant).

**Fig. 2:**
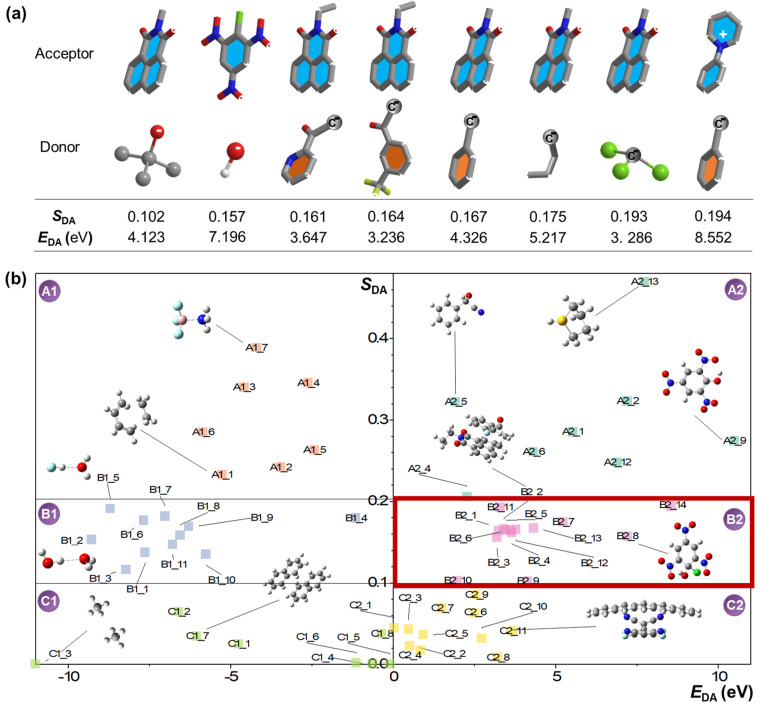
(a) Representative donor/acceptor pairs in the B2 region and their calculated overlap integral (*S*_DA_) and energy gap (*E*_DA_) values (in the order of increased *S*_DA_ value; hydrogen atoms are omitted for clarity except for hydroxyl ion). (b) Two-dimensional diagram showing where the calculated bimolecular interactions reside in arbitrarily divided regions: (A1) hard chemical reactions; (B1) hydrogen bonding; (C1) non-bonding interactions; (A2) easy chemical reactions; (B2) a hybrid interaction in between chemical reactions and single electron transfer; (C2) spontaneous single electron transfer. The chemical structures and calculated details for all the molecules are provided in the ESI.†

Does such a state exist? A positive *E*_DA_ value means that the state should at least consist a very strong donor or acceptor; a small but unneglectable *S*_DA_ indicates that the wave functions of the donor and the acceptor cannot contain too few or too many nodes at the same time, *i.e.*, one of them should be less noded like that of a lower atomic orbital and the other one more complicated, such as that of a π* or an atomic orbital with a high principle quantum number. We recently encountered a handful of molecules that exhibit bizarre physical properties which are hard to justify with traditional viewpoints. For example, we were unable to get any NMR signals for a series of pyridinium ylides,^25^ which are conventionally viewed as nucleophiles. Concomitantly, the electron paramagnetic resonance (EPR) experiment uncovered their radical character – although the intensity is one order of magnitude weaker compared to that of classical nitroxide or phenol radicals (Fig. 3a). Despite the identity problem, these ylides have been successfully used in diradical-mediated organic synthesis.^26^ Similarly, we also reported the discovery of unusually strong BMI between a carbanion (produced from a carbon acid such as acetophenone and a strong base such as *t*BuOK) and an *N*-substituted naphthalimide (Fig. 3b), the solutions of which not only exhibit vivid colors ranging from violet to green or indigo depending on the carbanion used, weak EPR spectra were also obtained with undiscernible NMR spectra. The system was first uncovered from failed attempts to conduct Claisen condensation reactions between acetophenone and ethyl acetate under basic condition, provided that *N*-substituted naphthalimide (Fig. 3c, NNI, >0.1 eq.) was present. For the binary system, we here also calculate the two parameters and find they fall right into the B2 region: *E*_DA_ = 3.664 eV, *S*_DA_ = 0.165. The common traits for the two systems are apparent: (1) the donor HOMO is higher in energy than the acceptor LUMO; (2) the donor wave function is quite simple and the acceptor complicated, which generates small but not near-zero overlap integral. Another recent example is from Eisenberg *et al.*,^27^ who found that when an ammonium salt is held close to an indole residual within a protein, the pair appeared to show a ground-state radical like state which is not obviously predicted by conventional knowledge. Although cation–π interaction is not uncommon, it is still puzzling to observed radical-like character between the pair and is hard to justify with non-bonding interactions such as electrostatic or polarization. However, when the present protocol is applied using the protein coordinates, we find substantial in-phase overlap between the C–N σ* and the indole π orbitals, although the *E*_DA_ value is slightly negative which may well be attributed to difference in solvent environment or computational method. Nonetheless, the small negativity can easily be overcome by thermal or photo-agitation to send the pair from B1 to B2.

**Fig. 3:**
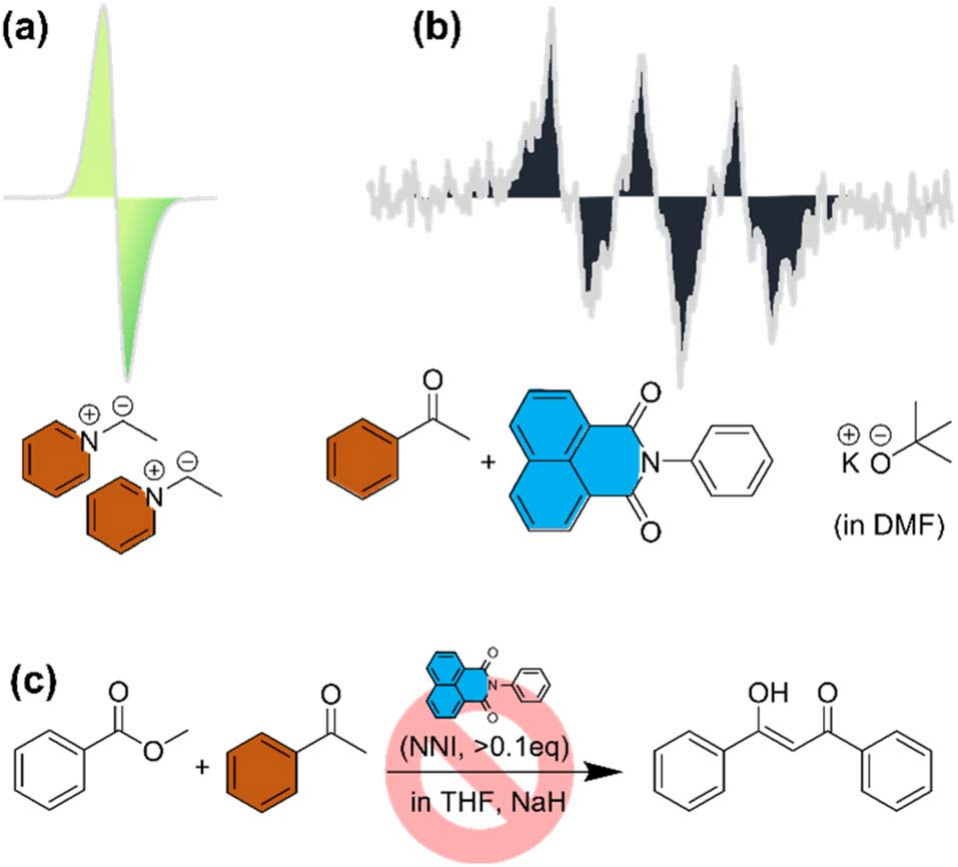
(a) Chemical structure of a pyridinium ylide and its EPR spectrum; (b) EPR spectrum of acetophenone and an *N*-substituted naphthalimide mixture in the presence of potassium *tert*-butoxide; (c) schematic illustration of a failed Claisen condensation when an *N*-substituted naphthalimide is present.

## Conclusions

In summary, the current study raises the possibility of systematically considering the CT component in bimolecular interactions using the molecular orbital theory, where two descriptors, *S*_DA_ and *E*_DA_, related to the frontier orbitals of a donor and acceptor can be used to generate a two-dimensional graph. The simplistic model treats the CT character of chemical bonding on par with that of van der Waals complexes, which is an unprecedented way of modeling a unified behavior among all molecular interactions. Nonetheless, it was found that similar types of BMIs are clustered near each other in this 2D-plot. Interestingly, a lesser studied region appears to be evident on this plot and is proposed as a hybrid BMI case between a conventional chemical bond and charge-transfer complex. We ascribe such a phenomenon to the mismatch between the numbers of nodes in the frontier orbitals when the energy match is satisfied: (1) small numbers for both create chemical bonding; (2) large numbers for both create CT complexes; (3) mismatch in nodal numbers creates a hybrid species, which is predicted to possess both stability and unpaired electron activity. The theorical study is also used to justify a few perplexing examples from recent studies from us and others.

## Conflicts of interest

The authors have no conflicts of interest to declare.

## Supplementary Material

RA-014-D3RA07729B-s001

RA-014-D3RA07729B-s002
