# Supplementary material for: 2D-Graph of intermolecular interactions predicts radical character of anion–π* type charge-transfer complexes
Source: RSC Adv. 2024 Jan 25;14(6):3771–5. doi: 10.1039/d3ra07729b (PMC10809263; doi:10.1039/d3ra07729b)
Supplement: RA-014-D3RA07729B-s002 [file RA-014-D3RA07729B-s002.pdf]

Electronic Supplementary Material (ESI) for RSC Advances.
This journal is © The Royal Society of Chemistry 2024

Supporting Information for

2D-Graph of Intermolecular Interactions Predicts Radical Character of Anion-π* Type of Charge-Transfer Complexes

Zhenda Lin, [†] *Hao Su,* [†] *Wenhuan Huang,* [†] *Xuepeng Zhang,** [†] *and Guoqing Zhang** [†]

[†]Hefei National Research Center for Physical Sciences at the Microscale, University of Science and Technology of China, Hefei, 230026, China

Corresponding Author

*zhangxp@ustc.edu.cn

*gzhang@ustc.edu.cn

1. Calculation

1.1 General calculation method

For all combinations, the theoretical calculation (including optimization, vibrational analysis and single point energy calculation) is taken at b3lyp/6-311+G(d,p) level with empirical dispersion(D3BJ). Among them, the combination at transition state, we checked that only one negative vibrational frequency exists, while the combinations without a transition state, none of the vibrational frequency is negative.

The overlap integral is calculated using the Multiwfn software pack (version 3.7) following the steps in the user manual(see 4.100.15 Calculate intermolecular orbital overlap integral of DB-TTF[1]), since the phase of (all)MOs in one certain molecular is arbitrary, the absolute value is used as the final result.

For some combinations with a 'cb-' notation in the A2 section, we made an imaginary reaction to form the molecular: the donor and acceptor moiety is placed at the actual position of the entirety, then the E_{DA} and S_{DA} is calculated as described above.

All of the calculation files can be accessed at: https://rec.ustc.edu.cn/share/697593f0-7043-11eb-9dc4-e13a4ed23fee

2. Results

2.1 Combinations in A1 section

Combination Name	Serial Number	Donor	Acceptor	$E_{DA}(in\ eV)$	S_{DA}	Explanation
TS-DA	A1_1	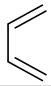		-5.263	0.233	[4+2], classic non-substituted Diels-Alder addition reaction
TS_AD-BH3_ene	A1_2	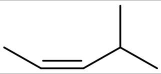	H_2B-H	-3.471	0.242	Transition state for an addition reaction
ct-NH3_BH3	A1_3	NH_3	BH_3	-4.595	0.340	Coordination of H_3N-BH_3
cb-BF3_NMe3	A1_4	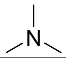	BF_3	-2.623	0.345	Coordination of Me_3N-BF_3
TS_AD-3_2	A1_5	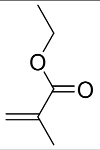	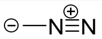	-2.433	0.263	Transition state for a [3+2] addition reaction, see *The art of writing reasonable organic reaction mechanisms*[2] page 225
cb-BOH3_NH3	A1_6	NH_3	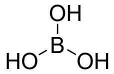	-5.858	0.285	The HOMO energy of NH_3 is low (at -0.27 a.u.), which results in a large negative value.
cb-BF3_NH3	A1_7	NH_3	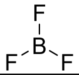	-4.213	0.389	The HOMO energy of NH_3 is low (at-0.27 a.u.), which results in a large negative value.

2.2 Combinations in A2 section

Combination Name	Serial Number	Donor	Acceptor	$E_{DA}\ (in\ eV)$	S_{DA}	Explanation
TS_AD-Et_ea	A2_1	$H_3C-\overset{\ominus}{C}H_2$	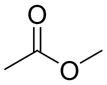	5.523	0.285	Transition state of a carbanion - carbonyl addition reaction
TS_AD-H2CF3Tone_EtNH2	A2_2	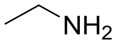	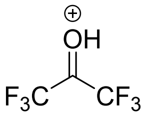	7.199	0.323	Transition state of an amine – activated carbonyl addition reaction
cb-ea	A2_3	$-O^{\ominus}$	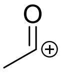	13.402	0.213	Reaction forming ethyl acetate from two ionic fragments
TS-RO	A2_4	$-S^{\ominus}$	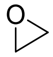	2.266	0.206	Transition state for the a ring-opening reaction
TS_AD-PhCHO_CN	A2_5	$\overset{\ominus}{CN}$	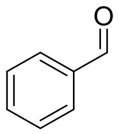	1.905	0.322	Transition state between cyanide anion and benzaldehyde
TS_AD-MeCN_Me	A2_6	$\overset{\ominus}{CH_3}$	$-CN$	4.309	0.261	Transition state for an addition reaction (see *The art of writing reasonable organic reaction mechanisms*[2] page 83)
cb_iPH3	A2_7	$\bar{P}H_2$	H^+	14.555	0.551	Ionic reaction forming PH_3
cb-2CF32T	A2_8	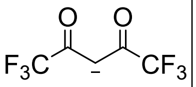	H^+	12.275	0.549	Neutralization reaction between an anion and a proton in vacuum

cb-3NO2Ar	A2_9	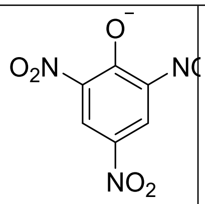	H^+	10.489	0.274	Neutralization reaction between an anion and a proton in vacuum
cb-4me6crNH	A2_10	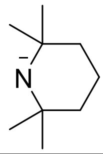	H^+	14.589	0.351	Neutralization reaction between an anion and a proton in vacuum
cb-5cAr	A2_11	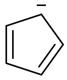	H^+	13.956	0.533	Neutralization reaction between an anion and a proton in vacuum
cb-5crOH	A2_12	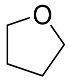	H^+	6.892	0.248	Neutralization reaction between a THF and a proton in vacuum
cb-5crSH	A2_13	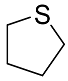	H^+	7.726	0.470	Neutralization reaction between a TFT and a proton in vacuum
cb-CH3OH	A2_14	$—O^-$	H^+	14.984	0.414	Neutralization reaction between an anion and a proton in vacuum
cb-F3CSO3H	A2_15	$F_3C-S(=O)_2-O^-$	H^+	11.508	0.358	Neutralization reaction between an anion and a proton in vacuum
cb-H2NPh	A2_16	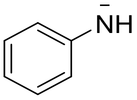	H^+	13.999	0.243	Neutralization reaction between an anion and a proton in vacuum
cb-H3PO4	A2_17	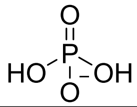	H^+	12.529	0.357	Neutralization reaction between a phosphoric acid and a proton in vacuum
cb-HCF3	A2_18	CF_3^-	H^+	14.925	0.672	Neutralization reaction between an anion and a proton in vacuum
cb-HCN	A2_19	CN^-	H^+	12.366	0.666	Neutralization reaction between an anion and a proton in vacuum
cb-itBuH	A2_20	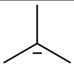	H^+	15.263	0.598	Neutralization reaction between an anion and a proton in vacuum
cb-iCH4	A2_21	CH_3^-	H^+	15.411	0.651	Neutralization reaction between an anion and a proton in vacuum

2.3 Combinations in B1 section

**Combination Name**	**Serial Number**	**Donor**	**Acceptor**	E_{DA} $(in\ eV)$	S_{DA}	**Explanation**
HB-CH4_NH3	B1_1	NH_3	CH_4	-7.635	0.137	Hydrogen bond between donor and acceptor
HB-H2O	B1_2	$H-O-H$	$H-O-H$	-9.283	0.153	Hydrogen bond between donor and acceptor
HB-EtOH_H2O	B1_3	—OH	$H-O-H$	-8.222	0.116	Hydrogen bond between donor and acceptor
HB-H2O_Cl	B1_4	Cl^-	$H-O-H$	-1.158	0.180	Hydrogen bond between donor and acceptor
HB-H2O_HF	B1_5	$H-O-H$	HF	-8.706	0.191	Hydrogen bond between donor and acceptor
HB-NH3_NH3	B1_6	NH_3	NH_3	-7.676	0.177	Hydrogen bond between donor and acceptor
HB-SH2_SH2	B1_7	$H-S-H$	$H-S-H$	-7.023	0.182	Hydrogen bond between donor and acceptor
TS_SN2-CH3Br_NH3	B1_8	NH_3	—Br	-6.553	0.158	Transition state in the S_N2 reaction between ammonia and methyl bromide

wNCBI-CH3Cl_CH3NH2	B1_9	$—NH_2$	—Cl	-6.294	0.169	Weak non-chemical bonding interaction
wNCBI-CH3Br_CH3NH2	B1_10	$—NH_2$	—Br	-5.762	0.135	Weak non-chemical bonding interaction
wNCBI-CH3F_CH3NH2	B1_11	$—NH_2$	—F	-6.787	0.147	Weak non-chemical bonding interaction

2.4 Combinations in B2 section

Combination Name	Serial Number	Donor	Acceptor	E_{DA} $(in\ eV)$	S_{DA}	Explanation
ours-NNI_2	B2_1	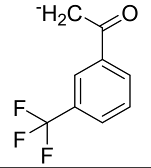	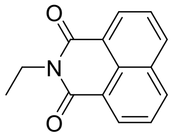	3.236	0.164	The lone pair on the carbanion is arbitrarily position to “connect” to the 4-position in the NNI derivatives
ours-NNI_3	B2_2	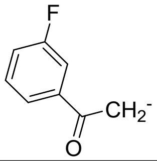	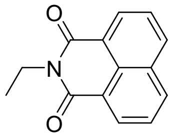	3.410	0.166	The lone pair on the carbanion is arbitrarily position to “connect” to the 4-position in the NNI derivatives
ours-NNI_1	B2_3	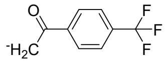	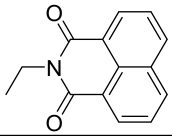	3.190	0.155	The lone pair on the carbanion is arbitrarily position to “connect” to the 4-position in the NNI derivatives
ours-NNI_4	B2_4	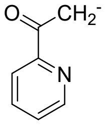	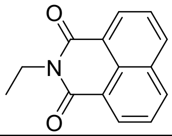	3.647	0.161	The lone pair on the carbanion is arbitrarily position to “connect” to the 4-position in the NNI derivatives
ours-NNI_7	B2_5	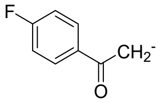	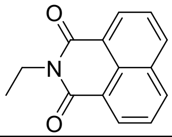	3.475	0.165	The lone pair on the carbanion is arbitrarily position to “connect” to the 4-position in the NNI derivatives
ours-NNI_9	B2_6	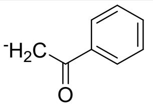	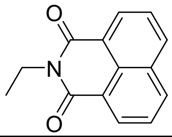	3.613	0.164	The lone pair on the carbanion is arbitrarily position to “connect” to the 4-position in the NNI derivatives
ours-NNI_nBu	B2_7	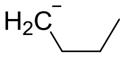	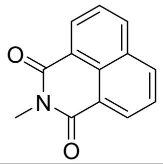	5.217	0.175	The lone pair on the carbanion is arbitrarily position to “connect” to the 4-position in the NNI derivatives
TS_SNAr-ClTNT_OH	B2_8	OH^-	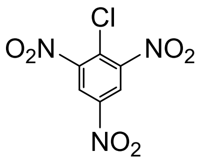	7.196	0.157	Transition state for the SNAr reaction, forming a sigma complex
ours-NNI_tBuO	B2_9	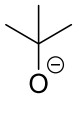	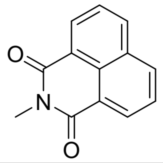	4.123	0.102	The lone pair on the oxide is arbitrarily position to “connect” to the 4-position in the NNI derivatives

TS_SN2-OH_CH3Cl	B2_10	OH^-	—Cl	1.966	0.102	Transition state for an S_N2 reaction
ours-NNI_CCl3	B2_11	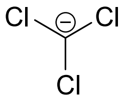	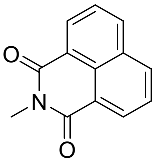	3.286	0.193	The lone pair on the carbanion is arbitrarily position to "connect" to the 4-position in the NNI derivatives
ours-NNI_Ph2C_stacking	B2_12	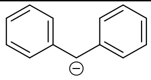	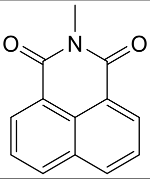	3.773	0.165	The lone pair on the carbanion is arbitrarily position to "connect" to the 4-position in the NNI derivatives
ours-NNI_PhC	B2_13	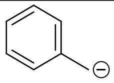	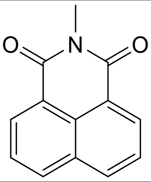	4.326	0.167	The lone pair on the carbanion is arbitrarily position to "connect" to the 4-position in the NNI derivatives
ours-PyPh_PhMe	B2_14	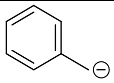	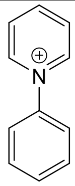	8.552	0.194	The lone pair on the carbanion is arbitrarily position to "connect" to the 4-position in the NNI derivatives

2.5 Combinations in C1 section

**Combination Name**	**Serial Number**	**Donor**	**Acceptor**	$E_{DA}(in\ eV)$	S_{DA}	**Explanation**
NCBIw-dimer_naphthalene	C1_1	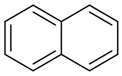	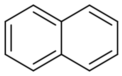	-4.755	0.0249	Weak non-chemical bonding interactions: π-π stacking
wNCBI-2PhH	C1_2	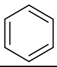	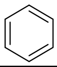	-6.607	0.0649	Weak non-chemical bonding interactions: π-π stacking
wNCBI-2CH4	C1_3	CH_4	CH_4	-11.016	0.0000301	Weak non-chemical bonding interactions: van der Waals force
cpi-NH4_HPh	C1_4	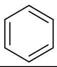	NH_4^+	-0.642	0.0000970	Strong non-chemical bonding interactions: cation-π interaction
cpi-Li_HPh	C1_5	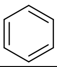	Li^+	-0.104	0.0000510	Strong non-chemical bonding interactions: cation-π interaction
cpi-Ar_menh3	C1_6	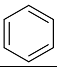	$—NH_3^+$	-1.138	0.00144	Moderate non-chemical bonding interactions: cation-π interaction
wNCBI-2PhBr	C1_7	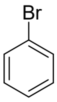	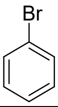	-6.040	0.0344	Weak non-chemical bonding interactions: cation-π interaction
ct-py_tcne	C1_8	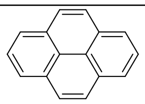	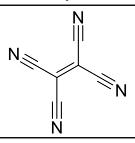	-0.354	0.0373	Strong non-chemical bonding interactions: cation-π interaction

2.6 Combinations in C2 section

Combination Name	Serial Number	Donor	Acceptor	$E_{DA}(in\ eV)$	S_{DA}	Explanation
ct-Tetracene_TCNQ	C2_1	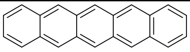	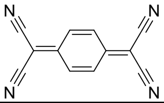	0.0235	0.0447	Charge transfer complex, charge transfers from donor to acceptor
ct-TTF_TCNQ	C2_2	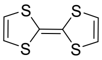	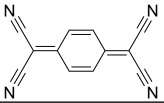	0.503	0.0223	Charge transfer complex, charge transfers from donor to acceptor
ct-per_4F2CN6Cr	C2_3	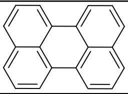	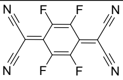	0.467	0.0425	Charge transfer complex, charge transfers from donor to acceptor
ct-pentacene_4F2CN6Cr	C2_4	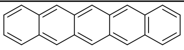	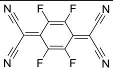	0.837	0.0171	Charge transfer complex, charge transfers from donor to acceptor
ct-DBTTF_4F2CN6Cr	C2_5	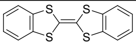	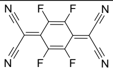	0.924	0.0360	Charge transfer complex, charge transfers from donor to acceptor
ct-2MeNPh_4NO22CN6Cr	C2_6	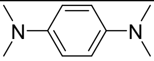	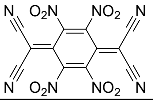	2.509	0.0627	Charge transfer complex, charge transfers from donor to acceptor
ct-2MeNPh_4F2CN6Cr	C2_7	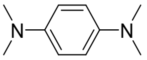	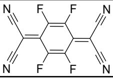	1.491	0.0690	Charge transfer complex, charge transfers from donor to acceptor
ours-NNI_PhO	C2_8	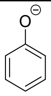	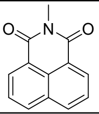	3.209	0.00834	Similar to the combinations in the B2 section, but the overlap integral is rather small
TS_E2-CH3Br_OH	C2_9	OH^-	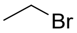	2.545	0.0853	This calculation suggests that OH- transfers an electron to acceptor's LUMO at the transition state during the E2 elimination in vacuum
ours-NNI_Ph3C	C2_10	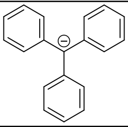	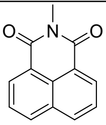	2.724	0.0317	Defined as "frustrated Lewis Pair"
PY_RCO	C2_11	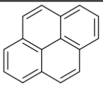	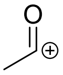	3.679	0.0406	A rather stable cluster configuration between donor and acceptor, which describes a stage in the Friedel-Crafts acylation

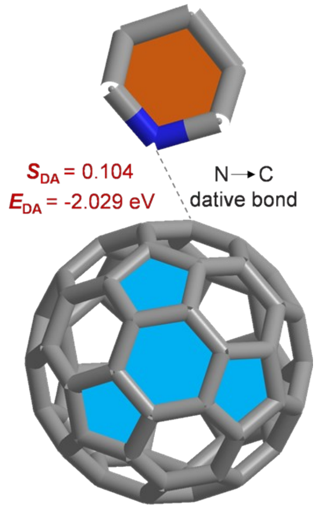

Figure S1. Calculated S_{DA} and E_{DA} values for the C_{60}-piperidine complex where a N→C dative bond exists as proposed by Hobza et al.

REFERENCES

[1] T. Lu, User manual for Multiwfn version 3.7, section 4.100.15, 2020

[2] R. B. Grossman and R. Grossman, The art of writing reasonable organic reaction mechanisms， Springer, 2003
